# Attitudes, Behaviors, and Risks of Sun Protection to Prevent Skin Cancer Amongst Children, Adolescents, and Adults

**DOI:** 10.7759/cureus.34934

**Published:** 2023-02-13

**Authors:** Jonathan R Raymond-Lezman, Suzanne Riskin

**Affiliations:** 1 Department of Foundational Sciences, Nova Southeastern University Dr. Kiran C. Patel College of Osteopathic Medicine, Clearwater, USA

**Keywords:** pediatric, adolescent, elementary, prevention, melanoma, skin cancer, sunscreen, spf, sun protection factor

## Abstract

Skin cancer is the most common cancer diagnosis worldwide. Many factors are involved in the development of skin cancers, but ultraviolet (UV) light exposure is the most modifiable. Our lifetime cumulative UV exposure may be a result of poor sun protection practices in childhood and adolescence. Modifying the attitudes and behaviors of children can be done in the classroom, at recreational activities, and at home. A review of the literature was conducted using Embase and PubMed to examine the relationship between attitudes and behaviors as they relate to sun protection strategies. Well-developed, standardized sun protection educational programs are needed to instruct children and adolescents. Individualized counseling from physicians or online programs is needed to help parents increase sun-safe standards for their children. Many young women utilize indoor tanning beds frequently, but many instructional programs increased their tanning bed usage. Time should be allotted at schools, sports practices, camps, and other outdoor activities for sunscreen reapplication. Many parents and children report the media is their primary source of information about sun safety. Media outlets may positively change attitudes and behaviors when reporting about sun safety. Parents and children need individualized programs or counseling to reduce ultraviolet (UV) exposure and increase sun protection. At-risk populations need tailored instruction, but few strategies have worked to decrease UV exposure.

## Introduction and background

Skin cancer is the most diagnosed cancer in the world. Incidence has been increasing since 2014 and is a global health concern for adolescents and children since many healthy activities and sports are conducted outside. Studies have indicated that sun exposure is the highest for individuals prior to 21 years of age [1]. It is estimated that 25-50% of ultraviolet (UV) exposure up until age 60 occurs during childhood It is also estimated that 25-50% of UV exposure up until age 60 occurs during childhood. Furthermore, it is estimated that a child receives three times the amount of UV exposure that adults do, annually [2]. Therefore, protecting children and adolescents is paramount to potentially lowering the incidence of skin cancer from exposure to UV light as a child.

Deciding how to influence attitudes and beliefs about skin cancer can be difficult. This can be attributed to varying platforms to teach, socioeconomic statuses, and variability in education. However, many approaches to protecting children from harmful UV radiation are achievable [3]. The best results from educating children about sun safety occur during grade school [3]. Reducing the incidence of skin cancer may be obtainable through behavioral changes in childhood, but designing strategies is difficult since most data regarding UV exposure in children is limited and mostly from interviews and surveys [2,4]. It has been suggested that schools are the best resource to implement changes and educate children about UV exposure [2]. A common theme is participants may be educated about the drawbacks of UV exposure, but knowledge alone is not enough to change sun protective behaviors. Peer pressure, unrealistic optimism where short-term gain outweighs the long-term risk, and a desire to look tanned and “healthy” all make patient behavior changes through counseling difficult [5].

Many current strategies to mitigate melanoma are tailored toward focusing on individuals with the lightest skin color and adopting a “one size fits all” approach. Standardized approaches to combat melanoma, such as wearing long sleeve clothing and sunscreen with a high sun protection factor, are important for individuals at an increased risk of developing skin cancer. While this is important to focus on high-risk populations, individuals with darker skin may experience confusion surrounding their melanoma risk [5]. Targeted, systematic approaches are needed to differentiate strengths and weaknesses that various sun-protective strategies work for varying skin colors. Maintaining serum vitamin D levels while inhibiting carcinogenesis involves multifaceted methods of protection. Few guidelines or studies exist which delve into this tiered approach to health.

Melanoma in childhood is rare. Only 2-3% of melanoma incidence occurs in children, but the risk should not be understated. Time increases the risk of developing melanoma with 85% of patients under 20 years, occurring between 15 and 19 years. One hypothesis is that genetic predisposition plays a greater role in melanomas that develop early in life [6]. This is compared to UV exposure which may have a greater role in developing melanoma later in adolescence. In a case-control study, there was a positive correlation between the number of sunburn episodes and the development of melanoma compared to controls. There has also been an association between a higher incidence of melanoma and latitude of residence during childhood. This suggests that UV radiation may impact adolescents similarly to adults in the development of melanoma. Genetic damage resulting in *BRAF* mutations was more common in patients diagnosed with melanoma at a mean age of 47 due to intense bursts of UV exposure early on in life. *NRAS* mutations were more common in patients with greater UV exposure during the ages of 50 and 60 with a mean age of 62 at diagnosis [4].

Glenn et al. stated, “Sun exposure is the primary modifiable risk factor for melanoma, with childhood estimated to be one of the most critical exposure periods for conferring risk”. Furthermore, first-degree relatives of melanoma survivors are at 8-12 times increased estimated risk of developing melanoma compared to individuals with no family history of melanoma. This is due to multiple factors, including shared sun exposure patterns, inherited predisposition, and phenotype [6]. However, the contribution of these risk factors in relation to the development of melanoma is not well-understood [6].

Many hypotheses emerge when attempting to determine the risk of developing melanoma. Some studies indicate that intense UV exposure during childhood may increase risk, but other studies have shown that the risk is similar if exposed to similar amounts of UV radiation in adulthood [7,8]. Further studies have concluded that some UV exposure during childhood may be protective against allergies by increasing serum vitamin D levels [9]. Additional studies are needed to determine the role of UV radiation and the development of skin cancer as well as genetic and environmental factors. This paper hopes to highlight many different strategies which have worked and not worked to increase sun safety amongst infants, children, and adolescents.

This article was previously accepted as a meeting abstract at the 2023 American Osteopathic College of Dermatology Annual Meeting and will be presented on February 22-26, 2023.

## Review

Aim

The goals of this review were to summarize findings from studies examining the behavior and attitudes of children, adolescents, and adults regarding UV protection. This review focuses on better education strategies, providing more resources, and determining the causes of inadequate UV protection. Finally, this review highlights areas where further research is needed.

Search strategy

**Figure 1:**
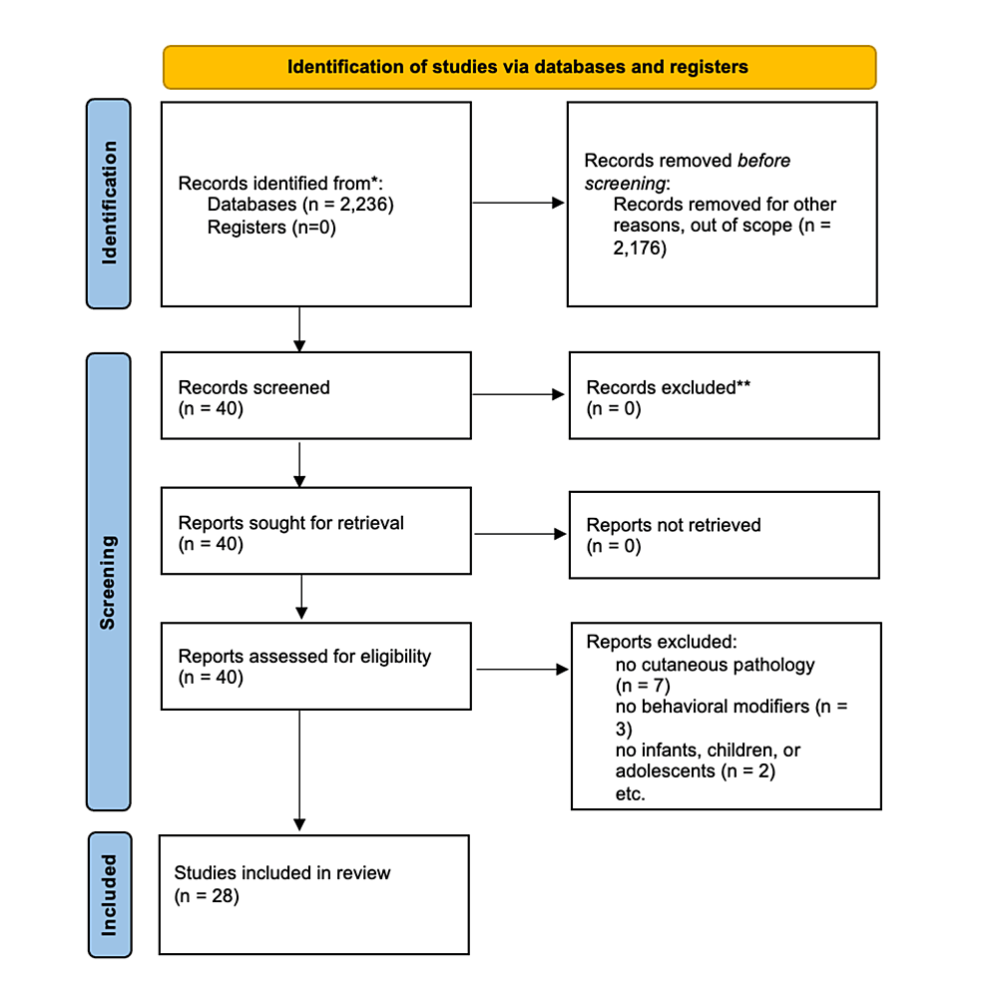
PRISMA diagram to determine studies used in behaviors and attitudes toward sun protection review PRISMA: Preferred Reporting Items for Systematic Reviews and Meta-Analyses

Two databases were used in the search: Embase and PubMed. Within Embase, the terms, “children AND vitamin AND d AND sun AND protection”, “(sunscreen OR sun) AND protection AND factor AND children AND cancer” were used. Boolean terms were used to limit articles that focused on the risks and benefits of UV exposure in childhood and adolescent populations. The terms, “sun protection factor children”, and “skin cancer children” were used in PubMed. A total of 2,236 articles were generated across Embase and PubMed. Articles that did not include cutaneous pathology, behavioral identifiers, or solutions to modify behavior, adolescents, or children were not included. After accounting for duplicate articles across the two platforms, a total of 28 articles were included in this review. Figure 1 demonstrates the selection of articles used in this review based on the inclusion and exclusion criteria.

Child, adolescent, and young adult UV exposure attitudes and behaviors

When Glenn et al. surveyed 336 parents, who survived melanoma, regarding their child’s sun protective strategies, the usage of sunscreen and wearing shirts with full length sleeves were both rated highly (78.5% and 75.08%, respectively). Wearing a hat when outside on a sunny day dropped to 29.91% and staying in the shade went down to 23.05%. Using sunglasses on a sunny day dropped further to only 8.41%. Parents indicated the total hours of sun exposure during a typical sunny weekend day also varied. Just 18% of parents said their child gets less than one hour of exposure daily, 49% said their child gets one to three hours of daily exposure, and 33% said their child gets four to six hours of daily exposure. When asked about sunburn, 43.03% indicated their child had at least one sunburn in the past year, and 9.6% of respondents’ children had a blistering sunburn at least once in the child’s lifetime. This study should be replicated with higher amounts of surveyed participants to gain more knowledge on perceived risks. This should also be looked at with scrutiny since it was a survey conducted over the phone, email, and mail-in [6].

Bivariate and multivariate analyses were also conducted to measure the psychosocial aspects of sun protection. The results indicated that higher usage of overall sun protection was found in younger age, female sex, having a higher objective risk, fewer barriers, higher perceived social norms of sun protection, and greater perceived efficacy of sun protection products. Ethnicity, time and staging at diagnosis, melanoma knowledge, family history, and perceived risk were not associated with overall sun protection [6].

Results from the study indicated that barriers are the greatest independent predictor of sun protection strategies. Age was a consistent predictor inversely related to the use of sunscreen, shirts with sleeves, sunglasses, and seeking shade. Gender was also separated where adolescent males were more likely to wear hats and shirts with sleeves. Adolescent females were more likely to wear sunglasses. This study indicated preliminary constructs that adolescents may use to combat sun exposure; however, it fails to determine if these are due to social constructs or from objectifiable perceived risk. More studies are needed to accurately determine the best methods adolescents may use to decrease their UV exposure [6].

Patel et al. conducted a study with 860 participants aged two to 18 years recruited from sports medicine clinics at large hospitals in California, Hawaii, and Colorado. A survey was conducted to assess the participants’ sun exposure behaviors and perceived risk during outdoor sports activities. Results indicated there was geographic preference for sun exposure and sun protection strategies. Sun exposure and tanning was the highest in Hawaii and lowest in California. Colorado’s participants used hats and sunglasses the most frequently. Sun exposure and burning risk also differed amongst females and males. While 42% of females perceived they sunburn easily if they did not wear sunscreen. Only 28% of males reported the same. Females also indicated they wear sunscreen, seek shade, use umbrellas to protect from UV light more frequently than males. Males reported wearing shirts covering the shoulders and hats more frequently than females [1].

Participants were categorized into four age groups. Participants who reported wearing sunscreen, hats, or protective clothing significantly decreased as age increased. However, wearing sunglasses and time spent in the sun to tan increased as age increased. Lighter skin color demonstrated positive correlation to wearing sunscreen, hats, sunglasses, shirts with sleeves, and clothing manufactured with sun protection material [1]. Since the study conducted was a survey, complete accuracy is unknown since surveyed individuals may indicate they utilize more protective barriers than they actually do.

Reyes-Marcelino et al. conducted a systematic review to procure data regarding sun protective strategies implemented at schools. Many of the studies examined were at high risk of bias. Sun protection studies were primarily conducted in elementary schools and 89% indicated positive change. Half of the studies indicated higher sun protection behavior after a four-week trial. Sunscreen usage increased in 73% of the studies examined. Shade seeking behavior increased in only 38% of the studies. Hat usage varied amongst different studies. While some reported higher usage of hats, others reported lower usage or no difference at all. The type of hat also influenced results where baseball cap-style hats decreased, and wide-brimmed hats increased. Studies examining sun protective clothing showed varied results with no identifiable conclusion. Almost every study which correlated self-reported sun behavioral changes, after indicating intent to change, showed no actual behavioral changes were made two to four weeks after the surveys were taken. A total of 87% of studies reporting effectiveness of increasing knowledge of sun exposure indicated participants effectively increased knowledge about sun safety [2].

In one study, high school students were less likely to increase their knowledge as compared to elementary school students. In 33 studies, attitudes toward tanning showed a 50% decrease in the desire to tan. Secondary school students remained the highest percentage of students who retained the desire to tan. These studies indicate that changing behaviors in adolescents is multifactorial and can be difficult to achieve. The results should be examined carefully due to the potential bias in surveys and confounding results. They provide details that the potential to influence young children is best done at the elementary level. More studies are needed to determine if the changes introduced early on in life continue throughout adolescence, teenage years, and beyond grade school [2].

Since teenage brains are not fully developed, many teenagers seek quicker gratification with little regard for the future. Vitamin D is synthesized through the skin via UV light absorption and helps modulate serotonin levels. This positive mental health aspect of UV exposure may influence adolescents’ views of increasing UV exposure to decrease depression. This can also lead adolescents to seek tanning behavior for aesthetics but not consider health ramifications in the future. Tanning is defined as purposeful intent to increase UV exposure to darken the skin. This can be accomplished through indoor UV tanning beds and through outside sun exposure. Adolescents who tan may do so for perceived health benefits. A study in Spain indicated that adolescent females who were dissatisfied with their appearance were strongly influenced by social media and outside pressures. They increased tanning behavior directly, or indirectly, from peer pressure. For many adolescents, exposing themselves to UV light, whether from a tanning bed or outside in the sun, is primarily done for darker skin [10].

Tanning may increase dependence on tanning by biomechanical mechanisms which release endorphins and increase tanning-seeking-behavior. A study with 1275 adolescents aged 14 to 17 years indicated that of individuals with indoor UV tanning history, 40% felt it would be difficult to quit. Furthermore, 45% thought they were unattractive without tanning, 9% attempted to quit but were unable to, and 21% had feelings of regret after tanning. In another study with university students who tanned, 30% neglected responsibilities and 14% skipped studying or working to tan instead. The growing desire for young people to want to tan is complicated and difficult to change. Since tanning behaviors are highly susceptible to social status, cues, and stigma, changing social dynamics is imperative to change behavior [10].

A questionnaire was conducted by Thoonen et al. to measure demographics, children’s sunburn history and extent, and parental sun protection behaviors. A total of 1,299 individuals completed the questionnaire. Results indicated that 42.4% of the children had at least one sunburn the previous year. Children with higher skin sensitivity and older children who were greater than 10 experienced more sunburn. Younger children and children with sensitive skin reported higher rates of sun protective strategies such as sunscreen application, seeking shade, and wearing protective clothing. Sunscreen and seeking shade were more often performed when outdoor activities were planned first. The rate of wearing protective clothing was higher when outdoor activities were not planned first. Socioeconomics may have influenced sun protective strategies. Most of the parents were middle-aged with most having Fitzpatrick skin types II (quickly burns, sometimes tans) or III (sometimes burns, tans easily). The youngest children with the most sensitive skin had the best rates of sun protection and the fewest amounts of sunburns [3].

While education about sun protection has improved, several studies report that the practices of these strategies may not be sufficient. Failing to apply sunscreen 30 minutes prior to exposure, improper amount of sunscreen used, skipping sensitive body parts, and failing to reapply as instructed on the packaging are just a few examples of pitfalls many parents face. This study addresses the need for more education on proper sun protective strategies. While it is positive that young children with sensitive skin are receiving protection, there is greater need to educate parents and ensure children of all skin types are adequately protected from UV light.

The risk of developing skin cancer is increased in pediatric populations with a history of malignancy. UV protection is of utmost importance to protect this vulnerable population, but adherence is difficult to determine. A study was conducted which compared 143 pediatric patients with malignancy to pediatrics without malignancy to determine if there are differences in sun protective strategies [11].

Results indicated that patients with malignancy spent less time outdoors and experienced less sunburn than controls. Sun protection strategies differed. Patients with malignancy were more likely than controls to always or frequently wear a hat in the sun (34.5% vs 20.7% p=.009). However, there were no differences between the groups in frequency of sunscreen use, wearing a shirt that covered the shoulders, seeking shade, or wearing sunglasses. There was also no correlation between sun protection habits and duration of sun exposure. Within the patient group and control group, religious participants had significantly lower sun protection as compared to non-religious participants [11].

Patients still undergoing treatment were less likely to spend time outdoors as compared to patients who completed therapy (83±83 minutes vs. 108±85 minutes). The time elapsed from diagnosis was positively correlated with time spent outdoors each day as well as UV exposure solely for tanning. These durations increased the greatest three years post-diagnosis. Time elapsed from diagnosis was negatively correlated with seeking shade but not with any other sun protective strategy [11].

Age did not correlate with time spent outside, but it was positively correlated with the number of sunburns per year and sun exposure solely for tanning. Age was also negatively correlated with sunscreen use, wearing a hat, seeking shade, and overall sun protection [11].

In another study, interviews were conducted by Kirk and Greenfield at a university to assess students’ attitudes toward UV tanning outdoors, UV tanning indoors, non-UV tanning such as sunless tanning, and sun protection. A total of 15 students aged 18 to 22 years were interviewed. A total of 80% indicated they prefer to be tanned, 53% attempted to tan while using sun protection, 53% used fake tans, and 20% had used indoor UV tanning [12].

Five main themes emerged. The students’ knowledge of UV radiation, sun protection practices, attitudes toward tanning, external influences, and internal influences. All the students indicated they knew that UV radiation from indoor tanning was associated with an increased risk of developing skin cancer. Many students indicated that feeling pain, a reduction in their activity, and how sunburns appeared on the skin were the main factors in preventing sunburn. Using sunscreen was considered a chore, an added expense, and uncomfortable. Participants agreed that sun protection strategies were mainly accomplished when traveling abroad [12].

Attitudes toward being tanned were mainly rooted in self-image. Tanned skin offered aesthetics such as appearing slimmer, covering skin defects like scars, and improving complexion. Drawbacks to tanned skin included not wanting to be too dark and not wanting an orange or patchy appearance from sunless tanner or indoor UV tanning beds. It was perceived that having a ‘healthy glow’ was sought after because it indicated leisure, holiday, vacation, and health were achieved. The students suggested that having a tan implies the individual has time to get away and spend time outdoors vacationing. However, negative perceptions were noted when asked about maintaining a light skin color or being pale [12].

External factors both positively and negatively impacted the desire for tanned skin. While sun protective routines and knowledge of UV radiation were shared amongst family members, some indicated that it was commonly a competition to see who could achieve the darkest skin. Social constraints amongst peers also increased the desire for tanned skin [12].

Internal factors comprised of unrealistic optimism regarding skin damage. Many participants disregarded their knowledge of the harms of UV exposure as an imminent threat to their health. This attitude resulted in riskier sun behaviors especially when wanting to spend time with peers outside. Some participants thought to start with low doses of UV radiation until a ‘base tan’ can be achieved. Others used indoor tanning beds to accomplish this prior to going on vacation. This study evaluated the attitudes of young adults, but the sample size is small. Additional studies should be conducted with similar metrics to gauge behaviors surrounding more university students [12].

A survey was conducted by Dennis et al. with 162 participants to assess if tanning behavior was due to a lack of knowledge or awareness of the harmful effects of UV exposure. Of those who partook in the survey, 51% self-identified themselves as having fair skin, 46% indicated they had a medium skin tone, and 3% reported dark skin. When assessing attitudes toward tanning, 77% of males and 89% of females indicated tanned skin was very or somewhat important. Among male fraternity students surveyed, 58% admitted using indoor UV tanning beds. When female sorority students were asked the same question, 99% reported using tanning beds with 96% having over 10 indoor tanning sessions over their lifetime. Only 39% of males had reported over 10 indoor tanning sessions [13].

Self-reported skin color was not associated with a perceived importance of tanning. However, lighter skinned individuals who stated they burn easily tended to have more sunburns as a result of tanning more frequently. They believed the sunburn would turn into tans. Among those who thought highly of having tanned skin, sunless tanners were rated highly as well. Increased knowledge of the negative effects of UV-induced tanning did not correlate with lower rates of tanning. Participants were likely to continue sunbathing despite their knowledge [13].

Students who reported that tanning was important indicated they mostly tan for personal gain and not due to peer pressure. The personal preference to look tanned was more related to feeling attractive, covering imperfections, and looking slimmer. These attitudes correlated with sunscreen use. Only 12% reported using sunscreen. The highest subset of individuals who wore sunscreen was among those who reported their skin as dark (36%) as opposed to those who reported their skin as fair (8%). Of all methods to darken skin, sunbathing was the most popular since it was the cheapest option. Sunless tanners were considered safe and effective, but with added cost and labor they were not sought after [13].

Additional studies should be conducted across more universities to determine trends with tanning. Interventions that educate the participants would help assess if any changes in knowledge can change behavior and attitude amongst university students.

Growing evidence links sunbed use to development of melanoma, especially when sunbed use started during adolescence. It is estimated by a meta-analysis that individuals who started using sunbeds prior to age 3 had a 15% increase in melanoma risk as compared to individuals who had never used a sunbed. The Australian Melanoma Family Study was conducted across various latitudes to determine associations between early onset melanoma and sunbed use. A total of 604 cases and 479 control groups were included in the experiment [14].

Results indicated that females (24%) were more likely than males (8%) to use sunbeds. Tanning salons (83%), gyms (72%), private homes (60%), and beauty salons (55%) were the most common locations for indoor tanning. A total of 18% of controls and 23% of case groups reported indoor tanning. The median age for the first use of indoor tanning was 22 years, the earliest reported was 14 years for cases and 16 years for controls. The median total sunbed usage was the same throughout regardless of age of first use [14].

Compared to control groups who had never used indoor tanning, those who had were reported to tan easily, be female, and have lower lifetime ambient UV exposure. Indoor tanning use was moderately correlated with higher socioeconomic status, lower total childhood sun exposure, and higher amounts of lifetime sunburns which blistered. Leisure time outdoors, skin color, family history, and education level were not associated with indoor tanning [14].

Participants who used indoor tanning beds were 41% more likely to develop melanoma as compared to those who did not. There were correlations between earlier first-use of tanning beds and the development of melanoma. Using tanning beds greater than 10 times had twice the risk of melanoma as compared to those who did not use tanning beds. Furthermore, using tanning beds greater than 10 times led to a four times increase in melanoma diagnoses for participants aged 18-29 years than for individuals with melanoma diagnosed between 30-39. Around 76% of melanomas for the 18-29-year group were associated with tanning bed use. Only 13% of melanomas in the 30-39-year group were associated with tanning bed use [14].

For individuals below the median value of lifetime UV exposure, there was a five times greater risk of developing melanoma from greater than 10 sunbed uses. However, the risk was not increased for individuals with higher lifetime UV exposure but with the same amount of tanning bed sessions [14].

Epidemiologic investigations have focused on determinants of melanoma. Gefeller et al. states that “the total number of melanocytic nevi has consistently been identified as the strongest risk factor for melanoma” with sun exposure as the main environmental cause of melanocytic nevi [15]. A German study with children aged six to seven years was conducted to determine the effects of cumulative and intermittent sun exposure on melanocytic nevi. By focusing on the history of vacations in sunny locations and patterns of sun exposure, the effects of these variables could be distinguished [15].

The study evaluated 940 females and 958 males with a median age of six registered at the local health department and whose parents consented to the study. The children were given health examinations in which the total number of nevi were counted. The parents were subsequently interviewed and did not know any details of the study to prevent behavioral changes. The number of melanocytic nevi in children ranged from zero to 43 with three being the average. No nevi were detected on 17.6% of children, 15.6% had one nevus, and 4.2% had greater than 10 nevi greater than 2 mm in diameter. A total of 79.8% of children had been on at least one vacation in a sunny location in their lifetime, averaging five weeks of vacation per child. No gender differences were found in any vacation variable [15].

Results indicated that increased vacations increased melanocytic nevi in children. The greatest percentage increase in nevi was due to vacationing in southern areas of high UV exposure whereas there were no associations with nevi and northern locations of vacation. The number of melanocytic nevi also increased as vacation duration increased from zero to one week to eight or more weeks. There were also linear increases in melanocytic nevi in cumulative groups which also had three to five or more than eight weeks of vacation. Only crude associations regarding cumulative sun exposure in the southern region could be made [15].

Barriers to sun protection are multifaceted and may produce socioeconomic concerns based on cost alone. Sunscreen, protective clothing, and hats are individual cost prohibitive barriers. Permanent shade structures, education, and interventional programs are systemic barriers. Furthermore, children report forgetting to apply sunscreen, disliking the feeling of sunscreen on their skin, missing certain areas on their body, applying too little, and failing to reapply regularly [16].

Social structures may also be attributed to poor sun protection strategies. With the desire to “fit in” and increase self-esteem, risky sun behaviors may result in darker skin but an increased risk of skin cancers. Norwegian adolescents aged 13 to 15 years indicated they felt more attractive with tanned skin [16].

Targeting adolescent groups for sun protection education is difficult since studies have indicated that adolescents are likely to increase UV tanning with more education about UV damage. Additionally, adolescents who perceived UV exposure negatively thought they sunbathed less than those who thought UV exposure was good for their health. Results from the study indicated that both groups of adolescents sunbathed at the same rates [16].

Nearly 200 journals have indicated that humans receive around 80% of their total lifetime UV exposure during adolescence. Many UV dosimeter studies have been conducted, but few compare children to adults. A prospective study was conducted to determine if UV dosage is linear with time or if adolescents receive disproportionately higher amounts of UV exposure [17].

Results indicated the highest amounts of UV exposure were during sunny vacations and days off in the month of July. There were no significant differences in UV dosage for participants less than 20 years. However, there was a significant difference in the annual UV dose in teen years compared to childhood. There were also significant differences in exposure between females and males under 20 years of age with females receiving more UV exposure [17].

The study’s parameters included the number of days with risky behavior, sunbed sessions or hours of outdoor activities, and UV exposure during workdays. There were significantly higher days with risky behavior when annual UV exposure was higher with teenagers leading. Teenagers received around 75% of their UV exposure during days with risky behavior. There were no significant differences between age groups taking vacations in sunny areas. No significant differences were also seen amongst children and teenagers and annual UV dose, sunbed sessions, or outdoor activities or sports but there were significant correlations between those parameters and adults. It was determined that older teenagers received around 25% of the total UV exposure they would receive during their lifetime if they kept the same UV exposure practices. This suggests that UV exposure may be linear with time if outdoor activities are the same. If adults migrate indoors for work, and do not spend time outdoors except on vacation, then childhood UV exposure will be high for lifetime UV exposure [17].

A survey was conducted to determine sun protective behaviors among adolescent males. A total of 137 male drum corps members were surveyed. When asked about sunscreen use, 96% indicated they apply sunscreen daily and spend less than 10 minutes on it. Only 37.5% reported sunscreen reapplication every two hours while 71.4% cited sunscreen made playing their instruments difficult due to the greasy texture. Around half (57.9%) found sunscreen to run into and burn their eyes, and 50.4% thought it took too long to apply sunscreen [18].

When comparing males at all-male locations to males at co-ed locations, males at all-male locations were more likely to wear sunglasses outside. There were no significant differences in the willingness to use sun protection practices among the two groups. Neither group had significant differences in the rates of sunburn in the two weeks prior to completing the surveys. Over one third (38.1%) of participants indicated that putting their sunscreen next to their water bottle enhanced usage. One third of participants did not feel that sunscreen on the sideline increased usage. Many felt there was not enough time for everyone to reapply [18].

Interviews were conducted at a co-ed site with self-selected males (n=31) to determine sun protective strategies. Almost all (93.5%) brought sunscreen to the site. However, while 71% applied sunscreen every morning, the remaining 29% only applied it if they felt they were burning or applied it during breaks. Around 74% of participants reapplied during meals, every four to six hours [18].

Many males felt that tanned skin was a positive consequence of sun exposure. They frequently cited sunburn and skin cancer prevention as reasons to wear sunscreen. The most common reason to avoid sunscreen was cloudy weather, uncomfortable feeling on the skin, or if they would not be outside for prolonged periods. The most popular commercially produced type of sunscreen used was the aerosol spray, but the sunscreen towelette received the highest ratings overall. Most males (74.2%) liked the ease of use that the spray provided. Finally, when shown images of older men with mottled, sun damaged skin, 64.5% of males indicated they would increase sunscreen usage to prevent premature aging [18].

Adult and parental UV exposure attitudes and behaviors

Glenn et al. sent a survey to eligible melanoma survivors using the California Cancer Registry database. A total of 324 Latino and non-Latino white melanoma survivors were surveyed who had at least one child zero to 17 years old. Results from this study indicated that parents were well informed about melanoma with an average score of 7.05 out of nine. They also perceived melanoma as a severe disease, scoring 9.54 out of 10. However, only 46% discussed their child’s risk for melanoma with the child’s physician and only half believed their children were at a higher risk for the development of melanoma. The other half felt their children were at the same or decreased risk of developing melanoma as compared to their child’s classmates [6].

A survey was conducted to assess sun protection awareness and behaviors of mothers with children under six years old. A total of 107 participants were included in the survey. When asked about time spent outdoors with their child, 72% of respondents indicated they spend seven days per week outside. For 64.4% of the respondents, more than one hour was spent each day outside [19].

Out of the 107 surveyed, 105 stated they apply sunscreen to their child. However, there was variation amongst that group regarding the type of sunscreen used. The sun protection factor (SPF) value was important to 87.6% of mothers while 51.4% chose the sunscreen based on its ingredients. Mineral filters were the most popular choice with 44.8% of respondents preferring them as compared to 1.9% who used chemical filters and 15.2% using mixed filters. An SPF of over 50 was important to 54.3% while SPF of 30 was preferred by 33.3% [19].

An important factor in sun protection is not only the ingredients and SPF of the sunscreen but also the application. Many different strategies emerged from the mothers surveyed. Before their walk, 37.2% of mothers who use sunscreen indicated they only apply sunscreen to their child’s face. In addition, 29.5% of respondents stated they only apply sunscreen once before the walk while 33.3% stated they apply frequently and as needed depending on the walk duration and weather conditions. Unfortunately, 14.3% of the children experienced erythema despite wearing sunscreen [19].

Age was a significant factor when determining if sunscreen was applied. Children under one year had sunscreen applied as indicated by 78% of mothers. However, only 49% of mothers with children greater than two years old applied sunscreen. This also correlated to the year-round use of sunscreen. While 21% of children less than two years old had sunscreen applied year-round, only 5% of children older than two had sunscreen applied year-round [19].

These statistics need additional studies to gauge respondents' knowledge of sun protection and perceived risk to understand better why there are wide differences between sun protection practices. The statistics are prone to bias due to the format as a survey and should be analyzed with scrutiny. Additionally, these statistics may indicate potential lapses in education standards, which may lead to an increase in UV exposure among adolescents [19].

A study was conducted by Littlewood and Greenfield utilizing interviews of parents with children less than five years old. It was structured, and the parents were recruited from local nurseries. Five of the participants had children with Fitzpatrick scale IV to VI skin color, which is darker. They tested four merits: attitudes toward sun protection in children, sun protective behaviors, sun safety knowledge, and motivating factors. Results indicated that all parents, regardless of their child’s skin type, felt that sun protection was important and took appropriate sun protective strategies. However, there were varying amounts of concern with some parents becoming increasingly anxious about UV exposure and other parents only exhibiting moderate concern. Parents also varied in attitudes toward tanning. One parent suggested that having a tan made you appear healthier, but also acknowledged that this was most likely a societal norm engrained within her. No parent wanted their child to be as tanned or tanner than they would be. Tanning was deemed an unimportant factor since positive tanning attitudes existed. There was no overlap with attitudes toward tanning and sun protective strategies. Knowledge of the risks of UV exposure also outweighed attitudes toward tanning [5].

Sun protective strategies were consistent amongst the parents with most admitting to using sunscreen, protective clothing, seeking shade, and limiting UV exposure. These strategies were most often implemented when on vacation outside of the UK, where this study was conducted. Parents stated that their vacation spots are much hotter with less cloud cover, so they predominantly use sun protection there but not at home. They perceive less risk with higher cloud coverage. They also stated that they tend to forget to put barriers on when at home even if they spent time outdoors during hot, sunny days. Parents discussed the disadvantages of sun protection. They found sunscreen was widely used but problematic due to the uncomfortable feeling it left on their skin. Parents of children with darker skin indicated sunscreen discolored their skin making it undesirable to use. Inorganic sunscreen which contains titanium dioxide or zinc oxide can leave a white haze when applied to the skin. While this type of sunscreen is safe and effective. However, since it is a physical UV blocker and sits on top of the skin which can leave residue, it may result in less sunscreen being applied due to negative attitudes toward the appearance it creates. Parents were split when discussing what type of sunscreen they purchased. Some parents indicated that they seek broad-spectrum “five-star” products, but others did not understand what sunscreen labels meant. Some parents lacked trust in the products and their efficacy [5].

Parents in the study recognized the importance of being a role model for their children. They also indicated that they often used less sun protection strategies than what they applied to their children. This was either due to the innate desire to be more tanned or from less perceived risk of skin cancer than their children’s risk [5].

When asked about skin cancer awareness, parents cited the media for helping with their sun safety knowledge. Attitudes toward their children’s risk varied amongst skin color. Some parents were correct in acknowledging that lighter skin with sunburn increases risk of skin cancer and darker skin lowers risk of skin cancer. Other parents felt there was no difference in the risk of developing skin cancer regardless of skin type. Social awareness impacted parents’ perceptions of UV exposure. Many felt it is frowned upon to have a tanned child [5].

This study, although small, highlighted many limiting factors to UV protection for parents and their children. Additional studies like this should be conducted in the United States and other countries to determine patterns of UV protection and attitudes toward UV protection.

It is widely known that sun exposure causes the skin to produce vitamin D. A cross-sectional study was conducted in Queensland, Australia to determine attitudes and behaviors toward sun exposure and vitamin D status. A total of 2,001 households completed the survey. The average age was 45, one-third had children under 13 years, and one-quarter obtained a university degree. Two-thirds of the households indicated they have fair skin and around one-quarter stated they tend to burn and not tan when exposed to the sun [20].

Most participants (84%) have heard of vitamin D before. The media was the most popular method of education (48%), then physicians (12%), and finally pharmacists (8%). Participants indicated in unprompted responses that vitamin D was beneficial for general health (22%), skin protection (20%), and bone health (14%). When asked if they regularly protect their skin from the sun and if they are at a risk of not getting enough vitamin D, 32% strongly agreed or agreed while 16% were unsure. Approximately 16% of participants planned to, and 21% of participants have already begun limiting sun protective behaviors due to concerns about vitamin D levels. Participants with a gross household income of less than $60,000 per year were the most likely to reduce sun protection and receive the highest doses of UV radiation [20].

Almost one-third (31%) of participants with children thought their child needed at least 30 minutes of UV exposure daily during the summer. A total of 43% stated their child needed at least 30 minutes of UV exposure during the winter. While 77% thought their children were maintaining adequate vitamin D levels, 12% thought their children were not maintaining adequate vitamin D levels with 16% of participants actively changing sun protective behaviors for their children. The greatest factors determining if sun protective strategies changed were if the parents had only earned a high school education, were in a lower-income household, had dark or olive skin, or parents who thought their children needed at least 30 minutes of UV exposure daily to maintain adequate vitamin D levels [20].

The media has the greatest influence on vitamin D knowledge across households. New sun-safe strategies should be implemented at news outlets highlighting the benefits of UV exposure while indicating sun protective strategies which do not lower vitamin D levels.

Melanoma incidence increased at a rate of around 3% per year. Children of melanoma survivors have a two-fold increase in the risk of developing melanoma if their parents had developed melanoma. A Los Angeles, California County cancer registry showed that 49% of melanoma survivors indicated that their children experienced at least one sunburn in the prior year. Sun protection interventions and education are important for this at-risk group and has more relevance and greater impact than the general public. A randomized controlled trial was conducted to assess psycho-social factors contributing to melanoma survivors’ children receiving adequate sun protection before and after an intervention was completed. A total of 2,014 melanoma survivors were screened and 340 ultimately completed the survey. Most (82%) resided in Texas or another southern state (10%) in the United States [21].

Results after the intervention indicated positive effects on children’s sunscreen reapplication after each hour spent outdoors. Children also wore more wide-brimmed hats before the intervention. The intervention had no observable effects on other sun protective outcomes such as seeking shade, wearing sun protective clothing, limiting UV exposure, or a composite sun protection score [21].

Children’s age is negatively correlated with sunscreen use and protective clothing usage. As child age increased, sunscreen use and protective clothing use decreased. Female melanoma survivors were less likely to change behavior after the intervention was completed. Family history of melanoma moderated the effects on time spent outdoors, but the intervention was less effective in families with melanoma survivors. Children’s sunburn incidence or shade behavior was unaffected by the intervention. Psychosocial outcomes, such as hats and clothing self-efficacy, clothing intentions, sun protection knowledge, and outcome expectations for their children all improved post-intervention [21].

As indicated by participants, the most efficacious method for behavioral change were DVDs (digital video discs) or booklets. Few participants indicated that their families also used the materials. More survivors from the non-interventional group indicated they received more sun protection information from non-study material sources. Interventional groups that used all the DVDs and booklets reported higher sun protection scores than survivors in the non-interventional group. Overall, intervention use was higher within survivors who had higher baseline levels of sunscreen and clothing use, limiting UV exposure, and overall sun protection score. Intervention was not associated with other survivor or child characteristics. More trials are needed to further enhance this study [21].

Understanding the sociocognitive factors which determine UV protective behaviors is important when strategizing future interventions. Many studies have indicated factors amongst adult populations, but only a few studies have compared behavioral factors between parents and their children. Studies that include this population usually focus on premotivational determinants such as attitudes, children under five years of age, sunscreen use as the main outcome, or knowledge of risk perceptions. While these results indicate the influence that sociocognitive determinants have on behaviors, more comprehensive insight is needed by investigating generic knowledge and behavior-specific determinants [22].

A longitudinal cohort study by Thoonen et al. regarding parental UV protection with four measurements was conducted. The online survey assessed demographics, execution sun protective behaviors, generic determinants, and behavior-specific determinants. A total of 670 parents completed the survey and were included in the analysis. Children of the parents surveyed were between four and 14 years of age. Self-reported measures of sunburn occurred at least once in 29.1% of the children during the previous summer. Sunburn at least once in a child’s lifetime was reported in 77.4% of participants. Sunscreen use was reported in 88.2% of parents [22].

Beliefs assessing attitude demonstrated the highest sample regarding sunscreen use. When assessing social norms there were positive associations with sun protection and parents’ willingness to discuss their partner’s opinions. Self-efficacy demonstrated the lowest sample means compared with other determinants. Clothing and shade-seeking behavior scores implicated room for improvement. When high difficulty completing a sun protective strategy was perceived, such as seeking shade, the lowest scores were depicted. Action plans contributed to the lowest sample means with the highest opportunity for improvement with seeking shade being the lowest scoring factor [22].

Generic determinants, such as knowledge, displayed high scores across all behaviors whereas risk perception displayed lower scores. Anticipated regret varied. Regret toward skin cancer development scored higher than those concerned about sunburn. Attitudes toward tanned skin remained low [22].

Since the 1980s, several studies have linked UV exposure during the first 10-20 years of life as a significant risk factor for developing melanoma and non-melanoma skin cancers. Therefore, sun protection should start early in childhood. When evaluating parents’ behaviors to protect their children, Zinman et al. reported that 91% of parents would employ at least one protective strategy. Interestingly, parents use of sunscreen with at least a sun protection factor of 15 resulted in fewer sunburns in their children. This increases evidence that parents as role models goes beyond socialization skills [23].

A study with 508 mothers in Queensland, Australia was conducted to assess knowledge, behaviors, sun protection, and sun damage. Surveys were conducted and self-reported measures were taken. An overwhelming majority (92%) of surveyed mothers indicated they understood they lived in an area with high incidence of melanoma, the sun was a causal link for developing skin cancer, and they needed to protect their skin from UV radiation even in the winter. Around 18% did not agree that sunburn caused harm to the skin [23].

The caregivers surveyed indicated that sunscreen was the least preferred method of sun protection with only 64% using it regularly on their child. Nearly all (93%) put their child in the shade, most (80%) put a hat on their child, and many put protective clothing (77%) on their child [23].

Attitudes toward sun protection were in-line with mothers’ goals. If the mother intended to absorb UV radiation for tanning, the child was less likely to receive sun protective strategies. Additionally, multiple factors for protection were added in series with one another. For example, if children wore protective hats, then they were significantly more likely to be placed in the shade (p<.05) and wear sunscreen (p<.001). Overall, logistic regression analysis revealed the greatest determining factor for UV protection for children was their mothers’ own selective sun practices most likely used herself [23].

Although public health messages have increased education about skin cancer and the effects of UV radiation, having tanned skin is still preferred in many countries. A population-based survey with 2,619 parents of children aged three to six years was conducted to examine attitudes and behaviors related to tanning and skin cancer. The average age of surveyed mothers was 34.3 ± 4.6 and the average age of surveyed fathers was 36.9 ± 5.3 (mean age ± standard deviation). In 65% of the children their hair was fair. In 68% of the children their eyes were either blue or green. There were no differences between the genders regarding distribution of photosensitivities [24].

Results indicated that parents who agreed that “tanned skin is healthy skin” had the lower level of knowledge. Those parents also had the lowest levels of sun protection at the beach for themselves and their children. There was no significant relationship between “tanning makes me look better” and knowledge. Parents who scored higher on knowledge also used more preventative sun protection for themselves and their children at beaches and at home while gardening [24].

Since this study was conducted via survey, there is potential for bias on behalf of those surveyed to indicate they use more sun protection strategies that they currently actually use. Furthermore, without knowing how the questions were asked, individuals surveyed who obtained less education than other individuals with advanced degrees may not have understood the survey in its original intent. However, it is important to note that knowledge of UV exposure and the dangers associated with it may directly correlate with increased incidence of melanoma. More public health strategies should be implemented which focus on parental education so at-risk populations of adolescents are adequately protected.

Parental behavioral factors also determine pediatric sun protective strategies. Parents report a lack of time, lack of interest, and a lack of counseling from pediatricians may be attributed to underusage of sun protection for their children [17].

Formal sun education programs and counseling benefits and pitfalls

With rising incidence of melanoma and non-melanoma skin cancers in adolescents, Germany introduced the SunPass programme in 2010. The intent was to reduce UV exposure in early childhood, raise awareness of sun exposure among educators, and reduce skin cancer incidence in the long term. A total of 40,000 children, 50,000 guardians, and 2,500 kindergarten educators completed the program from 2019 to 2021. The program contains lectures, inspections, certifications, and awards all quantified by survey before and after completion. The SunPass programme can be implemented as needed, following jurisdiction guidelines, and has been used in many other European countries [10].

A study conducted in Australia comprised of 426 students aged eight to 12 years assessed educational intervention and sun protection behavior. Researchers were given UV dosimeters, which measure UV radiation, and various sun protection methods. They were divided into groups of around four students per group. In various outdoor educational activities, students were asked to identify areas of the school that were sunny or shaded. With consideration of the time, season, and weather, UV indices were taken at various locations around the school. Students also tested various sun protective strategies to gauge effectiveness. These included: sunglasses, protective clothing, hats, and sunscreen. They were instructed to estimate the efficacy and then obtain UV measurements with the dosimeters. Students participated in interactive presentations about how UV exposure works along with risk factors for skin cancer and prevention. Students were also given a ten-question survey before and after the sessions which assessed UV and skin cancer knowledge [25].

The average pre-test total score was 6.3 out of 10 with a standard deviation of 1.4. The UV Index was poorly understood but a majority answered questions about peak UV time and ambient UV levels correctly. Health effects from UV exposure were moderately understood. After the exercises, the average post-test score was 8.9 out of 10 with a standard deviation of 1.3. The greatest improvement in knowledge was regarding UV index, but knowledge of all subjects increased post-exercise [25]. This study indicates the importance of educating students about UV exposure early on. While questionnaires are not foolproof, it prompts further research and strategic implementation of UV education strategies.

A systematic review was conducted by Henrikson et al. to determine if behavior counseling influences skin cancer prevention. A total of 2311 abstracts and 372 full text articles were reviewed. Out of these, 21 trials from 27 articles were used. A total of 16 trials were adult populations, and six trials were pediatric populations. Three adult trials were conducted using young adults (aged 17-25 or university students). All trials used parent-reported or self-reported behavior, skin protection methods, and sunburn [26].

Results for health outcomes from counseling indicated that among the six pediatric studies, counseling parents with children aged three to 10 years did not influence sunburn outcomes. In seven of the 16 adult studies, there was a decrease from 54.5% to 26.3% in reported sunburns three months after counseling. One trial (n=1356) indicated that there was no difference in the number of cancers or atypical nevi detected in interventional groups despite self-examination counseling [26].

Behavior outcomes from counseling parents of pediatric populations had no effect on interventional groups compared to controls. In adult populations, intervention produced mixed results. In one adult trial which used an interactive web program and five adult trials using tailored material such as text messages, mailings, or web programs, there was an increase in sun protective measures after completion of the program. Sunscreen use was the most influenced behavioral change. However, one trial found an increase in indoor tanning bed use among adolescent females. Of the 11 trials on skin self-examinations, nine indicated an increase in self-reported skin self-examinations compared to control groups [26].

Harms, such as increased UV exposure, vitamin D deficiency, reduced exercise, due to counseling were not reported in pediatric trials. In one primary care trial, the interventional group reported higher incidence of worrying about developing melanoma. The difference was not statistically significant [26].

The systematic review by Henrikson et al. increases awareness of the difficulties encountered with counseling patients. A multifaceted approach is needed to effectively educate patients on skin cancer, prevention, and treatment. Since few studies indicated behavioral changes from counseling, new strategies should be developed and implemented. Using online tools tailored to the patient leads to the best results. Indoor UV tanning among adolescent females continues to be a prominent health concern. Since UV tanning increased after counseling, more research is needed to determine what may positively influence this population to change behaviors. This study’s limitations, such as self-reported measures and surveys, show a growing need for more research into what counseling strategies work the best. However, this study highlights many positive outcomes and areas of improvement. Since counseling was primarily done in primary care settings, additional studies conducted by dermatologists would help identify potential strategies to improve sun protection behaviors [26].

Since many adult illnesses manifest due to behaviors during childhood and adolescence, the pediatric population should be targeted for melanoma prevention. Schools are optimal intervention sites since students often spend ample time outdoors and established education faculty and practices already exist. Few studies accurately present sun protective strategies used by children and adolescents. The most consistent conclusion is that children and adolescents are poorly protected from UV exposure. An assessment conducted in 2006 in Hillsborough County, Florida determined that fourth graders rarely wore wide-brimmed hats or shirts with long sleeves. Additionally, a study conducted in Palm Beach, Florida, found that only half of the students reported seeking shade most or all the time [27].

To aid schools, the Center for Disease Control (CDC) developed the Sun Safety for America’s Youth Toolkit. Additionally, research-tested interventions through Cancer Control P.L.A.N.E.T. aid cancer control planners locate effective interventions for preventing skin cancer. Through the National Comprehensive Cancer Control (NCCC) program, the CDC can assess the burden of cancer and implement plans to reduce incidence and mortality [27].

Three case studies using Comprehensive Cancer Control (CCC) partnerships, which utilized youth cancer prevention strategies, were conducted. The cases were conducted using semi-structured narratives provided by the CCC. Arizona, New Mexico, and Florida were three states included in the research. From 2003 to 2007, New Mexico and Arizona had non-statistically significant declines in melanoma rates, but Florida significantly increased. Each state used a similar Environmental Protection Agency (EPA) SunWise program but strategies for implementation varied across each state. Arizona sought 100% coverage of children attending public school. Florida demonstrated the least amount of coverage [27].

New Mexico started the RAYS program which used small grants sent to schools to implement sun-safety programs. It is guided by the socioeconomic model which recognizes the importance of individual behavior and change through policy and community actions. The RAYS program required participants to provide at least two presentations on skin cancer prevention using approved curricula. It consisted of educating students to avoid sun exposure between 10 am and 4 pm, use sunscreen with a sun protection factor of at least 15, and wear sun protective clothing. Self-administered pretests and posttests were conducted. Results from the posttests indicated that the program correlated with positive changes in behaviors amongst students and faculty [27].

Florida implemented the SPF Project at major schools. Its goal was to work with community-based programs at local health departments. The SPF Project uses the EPA’s SunSafe program material to address skin cancer for students who were in kindergarten through eighth grade (K-8). Schools that participate are granted a permanent outdoor UV protective structure to shield students from the sun. Several issues arose when the SPF Project was implemented. Many administrators were unable to take on the additional work of the project and sunscreen could not be applied in some districts since they considered it a medication. During the second year of the project, surveys conducted at schools indicated 80% of the schools allowed students to wear sunglasses and hats when outdoors. Two-thirds of the schools cited a need for more shade structures outside [27].

Arizona has the first mandated statewide sun-safety program. The goal is to teach Arizona children in grades K-8 how to reduce UV exposure, avoid sunburns, and prevent skin cancer. Their approach is comprehensive. It consists of after school partners, parks, and recreational programs which teach SunWise. Educators are trained, toolkits are supplied, and assemblies are conducted. The program collects data toward student attitudes and awareness regarding sun protective clothing, seeking shade, using sunscreen, and understanding the UV index. A total of 75% of the 1,455 students included in the study showed positive behavioral change after the program was implemented [27].

In 2003, the U.S. Prevention Services Task Force (USPSTF) determined that counseling patients in a primary care setting is insufficient to prevent skin cancer. Due to uncertainty surrounding whether counseling can change patient behavior, potential harms of sun-protective strategies, and limited fair, quality evidence linking sunscreen use or indoor tanning to skin cancer outcomes. A study was conducted in 2011 which identified gaps in the USPSTF’s original outline in 2003 to identify if counseling may reduce skin cancer. Investigation into 6,132 abstracts and 382 articles was conducted [28].

Results from the review indicated there were no trials that met their inclusion criteria for behavioral counseling and a reduction in skin cancer. There were 11 fair articles that discussed if counseling could help sun-protective behaviors. Results from these studies indicated that out of five trials, four showed modest improvement in sun protection behaviors with tailored counseling. However, the scores were small and may not be statistically significant [28].

Four trials conducted using an “appearance-based” approach to tanning prevention instead of a “health-based” approach showed positive influences from counseling. The average time spent under UV tanning beds decreased by 35% among women who had the intention of tanning indoors [28].

Two trials with children indicated a positive correlation between counseling and higher sun-protection scores. Parents of newborns also increased sun protective strategies when counseled compared to parents who did not receive counseling. However, the clinical significance of these scores is unclear due to the lack of statistical significance [28].

A study indicated that out of 484 schools surveyed, only 10% had sun protection policies. While sun education was prevalent, few offered substantiated protective measures for students [17].

## Conclusions

Children, adolescents, and adults are at risk for developing skin cancer when sun-protective strategies are not utilized. Counseling patients formally or using tailored educational tools can influence attitudes about UV exposure. However, behavioral changes were difficult to change. While some studies indicated positive behavioral changes after the intervention, most reported no statistically significant changes were made.

Education programs at grade schools may be implemented throughout all levels of education to increase sun-protective strategies and knowledge of ultraviolet light exposure. Formal programs in Europe have increased knowledge of sun protection and can be easily replicated. In the U.S., Arizona’s sun-safety program optimizes education from K-8 and can also be implemented in other school systems. Studies have indicated that starting sun exposure education for younger students worked better than when starting later as teenagers, but education for teenagers still improves sun protective strategies in some participants. However, teenagers were the least likely to implement sun protective strategies without a formal education program. Female adolescents are amongst the most difficult to evoke UV-seeking behavioral change, with some actually increasing UV exposure after counseling. Personalized counseling for patients is beneficial at nearly any age, but results may vary depending on socioeconomic status and educational achievements. The media plays a crucial role in delivering information on sun safety and studies have shown that it increases sun awareness and sun protection strategies. With an increasing incidence of skin cancer globally, more studies are needed to increase the knowledge of strategies needed to produce behavioral changes in individuals who seek the sun.
